# Effect of rhythmic auditory stimulation on gait kinematic parameters of patients with multiple sclerosis

**Published:** 2017

**Authors:** M Shahraki, M Sohrabi, HR Taheri Torbati, K Nikkhah, M NaeimiKia

**Affiliations:** * Department of Motor Behavior, Faculty of Sport Sciences, Ferdowsi University of Mashhad, Mashhad, Iran; ** Department of Neurology, Mashhad University of Medical Sciences, Mashhad, Iran; *** Department of Motor Behavior, Sport Sciences Research Institute of Iran, Tehran, Iran

**Keywords:** multiple sclerosis, gait, rhythmic auditory stimulation

## Abstract

**Purpose:** This study aimed to examine the effect of rhythmic auditory stimulation on gait kinematic parameters of patients with multiple sclerosis.

**Subjects and Methods:** In this study, 18 subjects, comprising 4 males and 14 females with Multiple Sclerosis with expanded disability status scale of 3 to 6 were chosen. Subjects were selected by available and targeted sampling and were randomly divided into two experimental (n = 9) and control (n = 9) groups. Exercises were gait with rhythmic auditory stimulation by a metronome device, in addition to gait without stimulation for the experimental and control groups, respectively. Training was carried out for 3 weeks, with 30 min duration for each session 3 times a week. Stride length, stride time, double support time, cadence and gait speed were measured by motion analysis device.

**Results:** There was a significant difference between stride length, stride time, double support time, cadence and gait speed in the experimental group, before and after the training. Furthermore, there was a significant difference between the experimental and control groups in the enhancement of stride length, stride time, cadence and gait speed in favor of the experimental group. While this difference was not significant for double support time.

**Conclusion:** The results of this study showed that rhythmic auditory stimulation is an effective rehabilitation method to improve gait kinematic parameters in patients with multiple sclerosis.

## Introduction

Multiple Sclerosis (MS) is a demyelinating inflammatory disease of the central nervous system that affects the gait ability, participation and quality of life of individuals [**1**]. About 85% of patients with MS suffer from gait difficulties [**2**]. Disabilities related to mobility as a result of gait limitations considerably restrict the patients’ activities of daily life and social interactions; hence, it has a major impact on the patient, family and community [**3**]. Although initial treatments of disease modulators decrease the rate of progression of disability by MS, which do not usually improve gait disorders [**4**]. These observations emphasize the need to develop interventions so that it can improve or stabilize gait in patients with MS. 

Rhythmic stimulation is an affordable, accessible and motivational method of gait rehabilitation. In some clinical populations, rhythmic stimulation and particularly rhythmic auditory stimulation (RAS) are employed to improve gait kinematic measures and motor unit firing patterns in some muscles associated with gait [**5**-**8**]. RAS is a neurological approach for improving the movement control in rehabilitation and treatment of physiological effects of auditory rhythms on the motor system [**9**]. It is also a technique rhythmic motor cuing to facilitate the training of movements that are rhythmic inherently and biologically [**10**]. The major mechanism of gait training with RAS is the auditory–motor synchronization in the central nervous system that reflects the auditory rhythm in the output of functional motor; for instance walking [**11**]. Auditory rhythm activates both motor and auditory areas of the brain [**11**-**17**]. Activation of motor areas brings about lower muscles activation and leads to better control of gait [**18**-**20**]. 

Gait disorder is well proven in MS [**21**] and is reflected in the spatial-temporal parameters and also in gait kinematics. These changes include reduced stride length, step length, cadence, gait speed and increased double support [**22**-**24**], which can be as a result of fatigue [**25**,**26**], reduced strength of leg muscles [**24**,**27**] and spasticity [**28**], lack of coordination resulting from cerebellar lesions [**29**] and the decline in attention [**30**]. 

A good number of previous studies on gait training with RAS in patients with neurological disorders have demonstrated the improvements in gait spatial-temporal parameters such as stride length, cadence, and gait speed [**10**,**31**-**34**]. However, to the best of our knowledge, only one of the pilots published studies has examined the effect of RAS on gait in patients with MS. In this study, the subjects received a RAS in-house training program aimed at improving gait function. The results obtained revealed a significant decrease in double support time between the groups. A pooled within-group analysis also revealed that stride length, step length, cadence, gait speed, and normalized speed have been significantly improved following a week of treatment program [**4**]. Although this study demonstrates the effects of potential gait training with in-house RAS on the gait parameters of patients with MS, it has limitations such as small sample size, and lack of exercise control by the researcher. Furthermore, in this study, the control group did not receive any exercise. The study authors suggested that other studies are required to review the performance of different protocols based on RAS in gait of patients with MS [**4**]. 

It seems that further studies are needed to support the utilization of RAS in patients with MS. Therefore, in this study, we tried to eliminate some restrictions in the previous study (sample size, lack of exercise control and lack of exercise in the control group). We also tried to examine the impact of RAS on the gait kinematic parameters of patients with MS by means of stimulation and more accurate measurement tools. 

## Subjects and methods 

In this study, 23 MS patients comprising 7 males and 16 females from the Khorasan Razavi MS society participated in this research by means of available and targeted sampling method. Inclusion criteria were: age range of 18 years or older, Expanded Disability Status Scale of 3 to 6, the ability to gait at least 100 feet without physical assistance, lack of treatment for relapse and exacerbation of MS in the past 30 days, lack of cardiovascular and rheumatism diseases, lack of severe pain in the lower joints, engaging in no regular physical activity in the past three months and the lack of hearing impairment. All subjects completed the study consent form. Subjects were randomly assigned into two experimental and control groups. Eighteen (18) subjects completed the study while 5 subjects were excluded from the initial sample in the study. General characteristics of subjects are presented in **Table 1**.

**Table 1:** General characteristics of subjects (n=18)

	RASG (n=9)	CG (n=9)
Gender (M/ F)	2/ 7	2/ 7
Age (yrs)	40.33 ± 6.67	38.11 ± 12.12
Height (cm)	162 ± 8.94	160.39 ± 9.70
Weight (kg)	63.72 ± 9.30	66.14 ± 7.24
RASG: Rhythmic Auditory Stimulation Group CG: Control Group		

In this study, the subjects from the experimental group performed the gait training with rhythmic auditory stimulation and by listening to the metronome beat. In the present study, a metronome device of Musedo (MT-100) brand was utilized together with a XP-HS825 headphone to create stimulation. At the beginning of the first session of the training program in the experimental group, the metronome output beat was set at 10% higher than the preferred cadence of each subject [**4**]. The 10-meter gait test at preferred speed was utilized for this purpose [**35**]. Subsequently, the subjects were asked to match their steps to the metronome output beat, and gait the 6-meter distance, rotate 180° and return to the trailhead [**36**]. Training was carried out for three weeks, with 30 min duration for each session, three times a week [**33**]. In each session, the time was shortened if the 30-minute gait was difficult for the participants. Moreover, for each week, the researcher ensured that the metronome output beat for each subject is in accordance with the preferred cadence plus 10% [**4**]. A coach accompanied each subject to ensure the accuracy of the training and to care for the subjects during the training. The subjects in the control group performed similar trainings as the experimental group without the use of stimulation.

Qualisys motion analysis device was utilized for measuring the subjects’ gait kinematic parameters. It was made in Sweden with eight cameras and QTM software. The preparation of the subjects was carried out after camera set up and calibration in the walkway space. 

Marker Placement was conducted according to Helen Hayes protocol, after explaining the measuring process to the participants. Thereafter, participants were asked to gait the walkway several times tentatively and with the aim of increasing the stability of the gait. Afterwards, gait was measured and recorded. The measurements were carried out before (pre-test) and after (post-test) training for all the subjects. All the measurements were conducted in the laboratory of motion analysis in the Islamic Azad University of Mashhad.

Data processing was carried out by utilizing MATLAB software version 2014. For information processing, the accuracy of the information was evaluated first. After reviewing the accuracy of the information, spatial-temporal parameters of stride length, stride time, double support time, cadence, and gait speed were extracted from the kinematic information. After calculating the variables of any trail recorded by motion analysis system, their average was calculated for each participant. SPSS version 18 was employed for statistical analysis. Data normality was examined by Kolmogorov-Smirnov test. To evaluate the within-group effects in two experimental and control groups, paired-sample T-test was utilized. The analysis of covariance (ANCOVA) was employed for the comparison of gait kinematic parameters between the experimental and control groups in the post-test. 

## Result

Within-group analysis for the experimental group demonstrated a significant difference between the mean scores of pre- and post-tests in parameters of stride length, stride time, double support time, cadence and gait speed (P < 0.05).

Within-group analysis for the control group demonstrated that there was no significant difference between the mean scores of pre- and post-tests in parameters of stride length, stride time, double support time, cadence and gait speed (P > 0.05). In addition, comparing the gait kinematic parameters in the post-test mode between the experimental and control groups by the analysis of covariance (ANCOVA) revealed that there was a significant difference between the parameters of stride length, stride time, cadence and gait speed in both experimental and control groups in the post-test mode after excluding the effect of pre-test (P < 0.05). However, this difference was not significant for the double support time (P > 0.05) (**Table 1**). 

**Table 2:** Post-test mode between the experimental and control groups by the analysis of covariance (ANCOVA)

	RASG				CG				
	Pre-test	Post-test	t	P value*	Pre-test	Post-test	t	P value*	P value**
Stride Length (m)	0.58 ± 0.11	0.97 ± 0.17	-6.08	0.00	0.75 ± 0.27	0.81 ± 0.30	-1.98	0.08	0.00
Stride Time (s)	1.65 ± 0.38	1.13 ± 0.22	4.45	0.00	1.32 ± 0.14	1.33 ± 0.22	-0.11	0.92	0.03
Double Support Time (s)	0.41 ± 0.15	0.19 ± 0.06	4.27	0.00	0.28 ± 0.09	0.26 ± 0.10	0.47	0.65	0.16
Cadence (steps/ min)	73.79 ± 21.26	109.54 ± 19.84	-6.09	0.00	91.69 ± 10.51	93.05 ± 15.37	-0.27	0.79	0.00
Gait speed (m/ s)	0.38 ± 0.16	0.90 ± 0.32	-5.43	0.00	0.57 ± 0.24	0.64 ± 0.30	-1.40	0.2	0.00
RASG = Rhythmic Auditory Stimulation Group; CG = Control Group; *P value t-test; **P value ANCOVA test.									

## Discussion

The results obtained from the present study showed that the gait training with RAS could have significant effects on the gait kinematic parameters of patients with MS, including stride length, stride time, double support time, cadence, and gait speed. These results were significant for the experimental group in parameters of stride length, stride time, cadence, and gait speed after the training, when compared to the control group indicating the positive effects of RAS on improving gait performance in patients with MS.

A good number of previous studies has demonstrated the effectiveness of RAS in patients with neurological disorders, improvements in spatial-temporal parameters such as stride length, step length, cadence and gait speed [**10**,**32**-**34**], which were in line with the results of this study. On the other hand, some studies on patients with Parkinson’s disease have shown that despite the significant increase in step length and gait speed, following the gait training with rhythmic auditory cue which is consistent with the results of this study, the cadence had no significant change [**35**,**36**]. This contradiction in cadence improvement can be justified by the fact that the temporal aspects of gait are limited to the frequency of the employed cue [**36**]. In the mentioned studies, rhythmic auditory cue was set in a frequency based on the subjects’ preferred gait cadence. However, in this study, it appeared that a significant increase in the cadence of rhythmic auditory cue frequency was 10% higher than the subjects’ preferred cadence. The results of this study supported the findings of Dwyer Conklyn et al. (2010) on the significant improvement in stride length, cadence, and gait speed in patients with MS [**4**]. The non-significant difference in the improvement of double support time in this study might be induced by training in control group.

Gait speed can be increased by increased cadence or stride length or both [**37**,**38**]. In this study, an increased gait speed was due to an increase in stride length and cadence. The movement timing error in the scheduled responses is decreased as a function of the increase in movement speed [**39**]. The natures of coordination derive from timing and the order of multiple muscle groups’ activation is different; therefore, discoordination can be due to problems in the timing of multiple muscle groups’ activation. Exercising a practical motion under applied time constraints can have effects on timing and the treatment of coordination problems in patients [**40**]. Moreover, a prolonged double support time in patients is related to a drop in gait speed and instability [**40**,**41**]. Therefore, an increase in gait speed and decrease of double support time as observed in this study might reveal the improved coordination and increased gait stability in patients with MS. 

The key concept of RAS is the auditory–motor synchronization in the reticulospinal tract [**11**]. A good number of studies have shown that auditory rhythm activates the motor areas of the brain including the supplementary motor area (SMA), pre-supplementary motor area (pre-SMA), premotor cortex (PMC), ganglia basal and the cerebellum [**12**-**15**]. The activation of the motor areas of the brain via the rhythm enhances muscle activation and leads to better movement control [**7**,**20**]. RAS can also be utilized as an external stimulation and have effects on the discharge of motor neurons as well as reducing the muscle fatigue and automatic movement reaction time; thus, it can improve the quality and delay of a special response [**10**]. 

It is important to maintain balance in gait [**10**]. Horak (1987) demonstrated that medial-medial geniculate nucleus of the vestibular system in the ears mainly affects the standing balance. When auditory stimulation reaches the organ of Corti, the signal is transmitted to the medial-medial geniculate nucleus and then reaches the auditory cortex in the temporal lobe. This activates the vestibular system to improve standing balance. Therefore, as an auditory stimulation, RAS can enhance the balance in this method [**41**]. 

Due to the central nervous system damage in MS patients, attention and memory capacity is reduced [**30**,**42**,**43**]. Therefore, the simultaneous focus on different symptoms is not possible for them [**44**]. Employing external rhythmic cues can enhance gait by guiding the attention to outside [**45**,**46**]. In terms of external attention, a smaller amount of load is applied to the attention resources or working memory, because the performer processes only a single source of information that is relative to the external performer, and therefore, it leads to better performance of movement [**47**]. Moreover, according to the limited action hypothesis, external attention improves a kind of automatic control, and decreases the conscious intervention of the movement control; as a result, it increases performance and learning [**48**,**49**]. External attention increases the coordination between organs, and the muscular system can control the action with less conscious control, and allows a smoother movement [**48**-**50**].

Gait natural rhythmic movements can be corrected via synchronization and external attention processes, which can result to the improvement of gait in patients with MS. In these patients, RAS may have a positive impact on the consequences of the disease such as lack of coordination [**29**], fatigue [**25**,**26**] and balance impairment [**51**].

## Conclusion

The results obtained from this study showed that RAS is an effective rehabilitation method for improving gait kinematic parameters in patients with MS.
